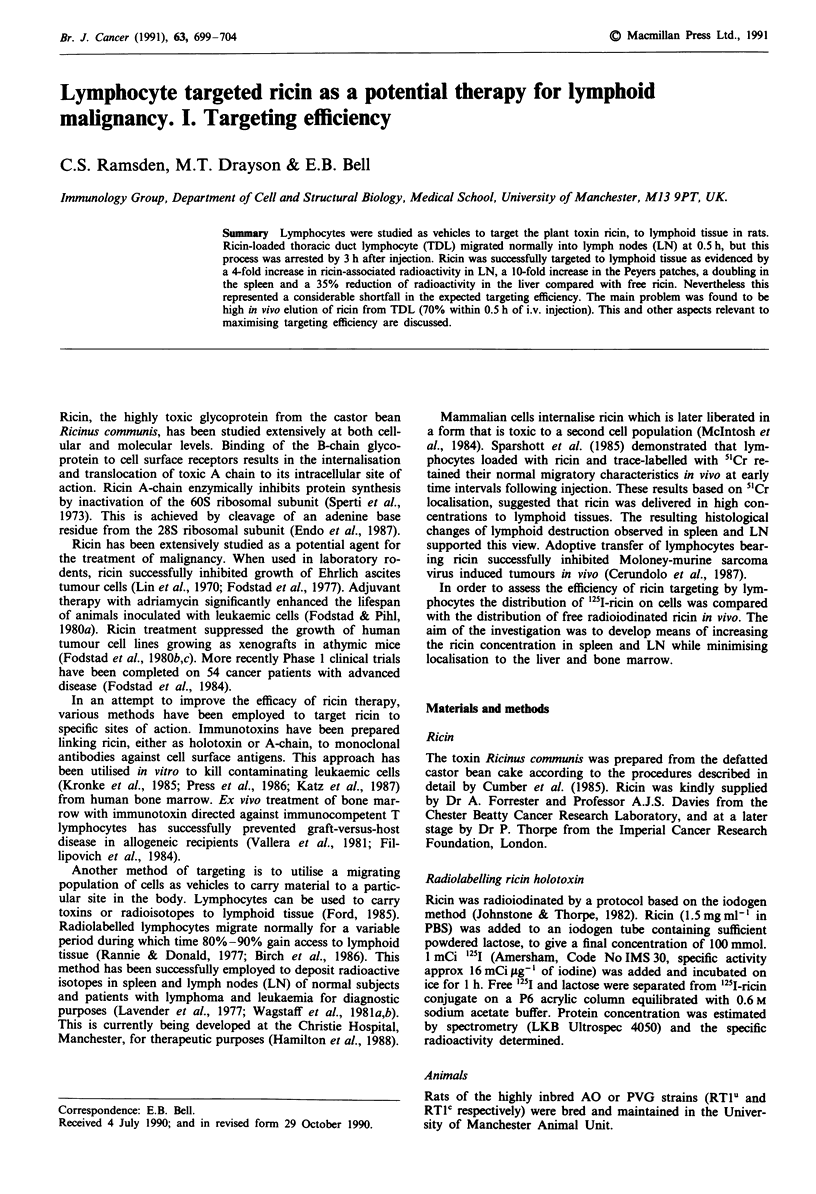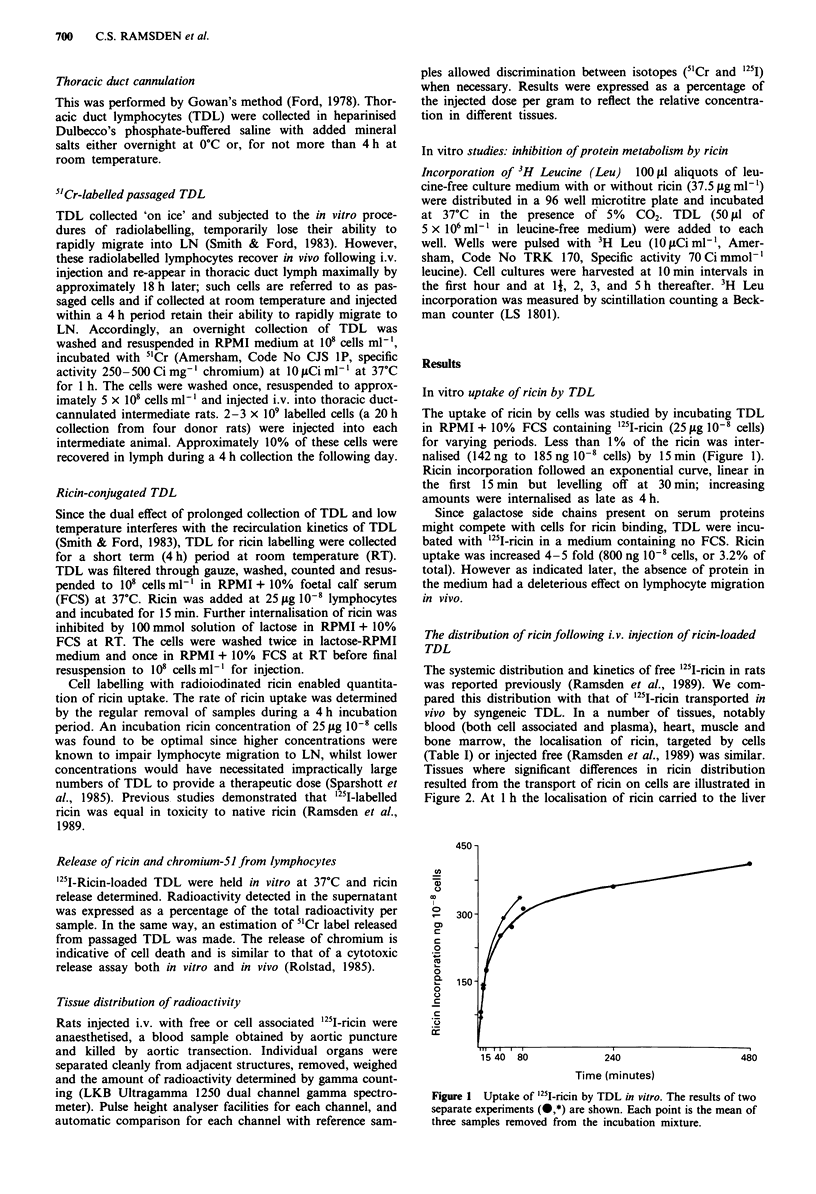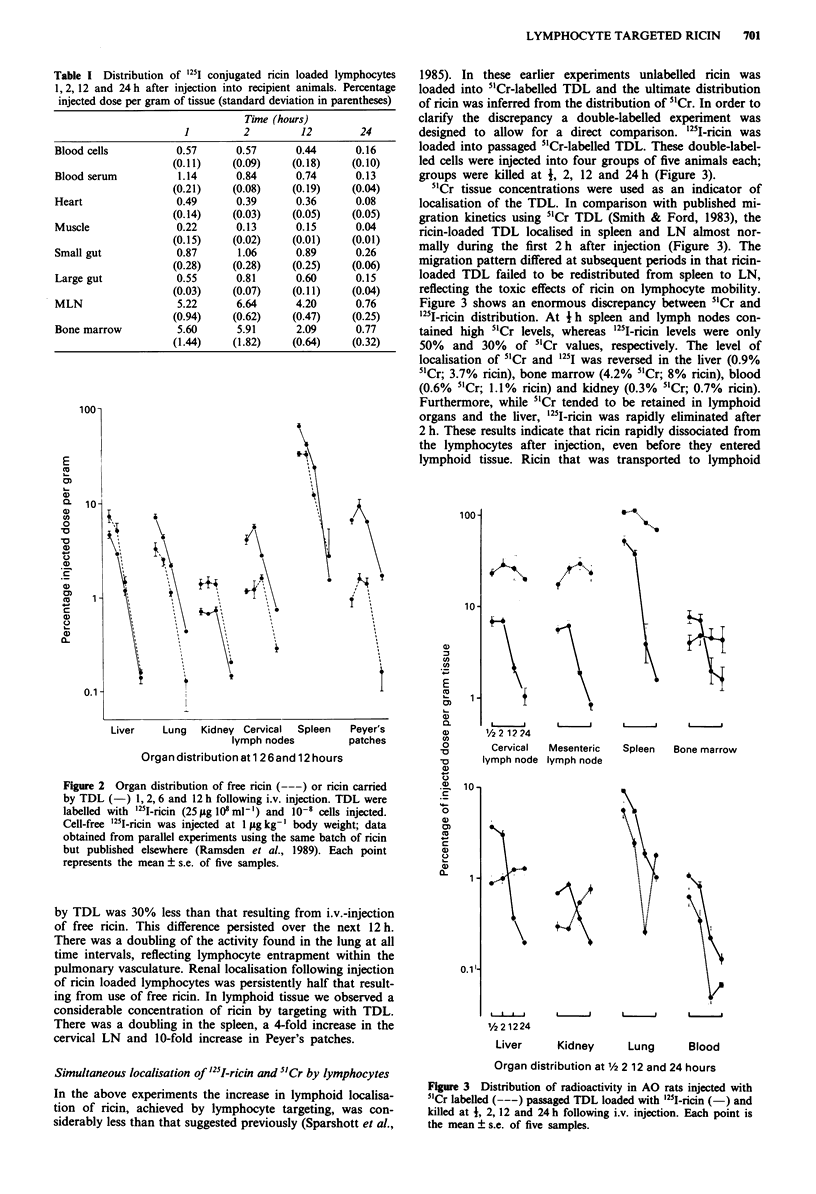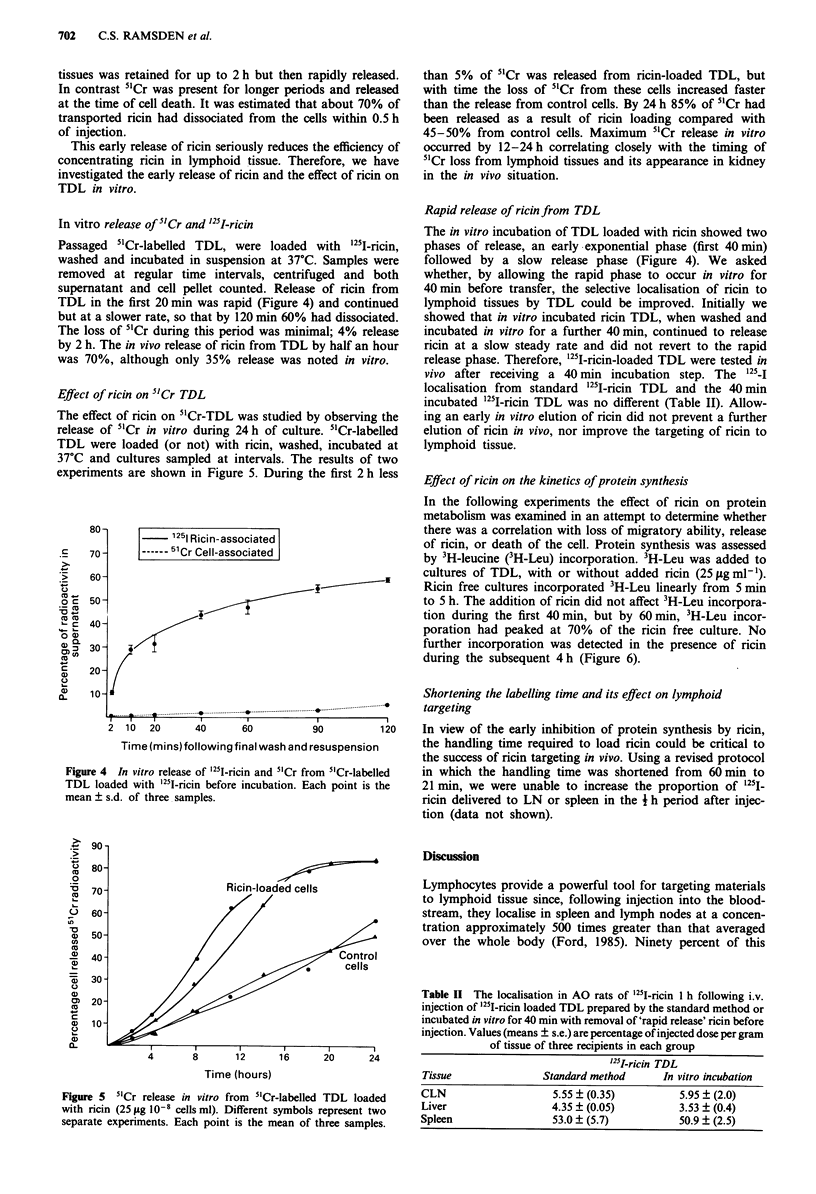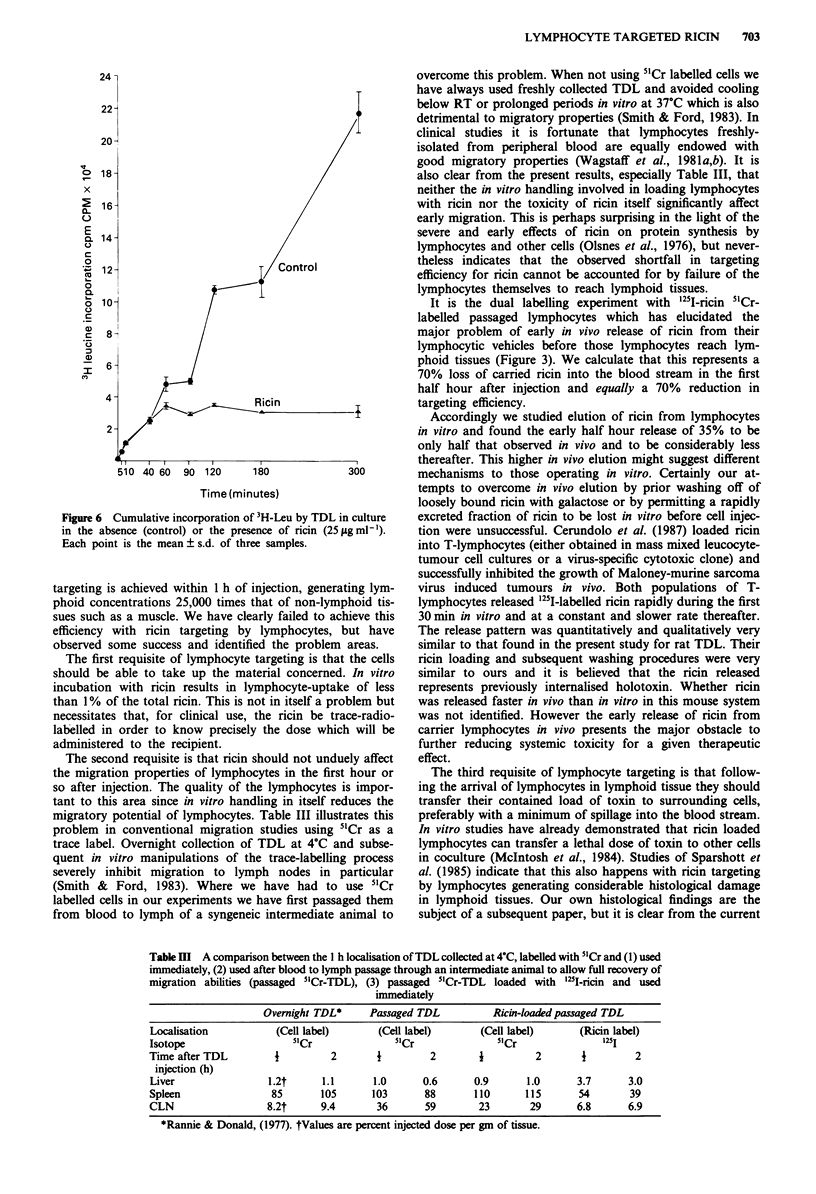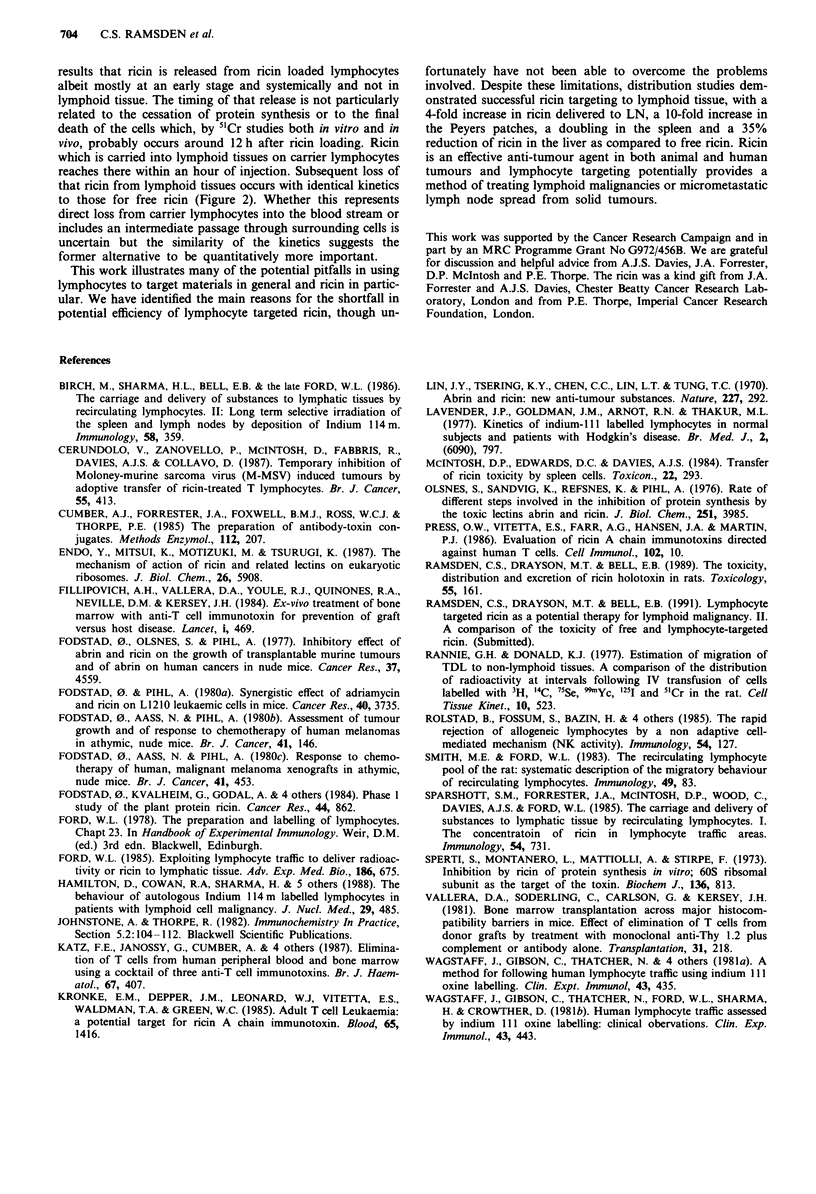# Lymphocyte targeted ricin as a potential therapy for lymphoid malignancy. I. Targeting efficiency.

**DOI:** 10.1038/bjc.1991.158

**Published:** 1991-05

**Authors:** C. S. Ramsden, M. T. Drayson, E. B. Bell

**Affiliations:** Department of Cell and Structural Biology Medical School, University of Manchester, UK.

## Abstract

Lymphocytes were studied as vehicles to target the plant toxin ricin, to lymphoid tissue in rats. Ricin-loaded thoracic duct lymphocyte (TDL) migrated normally into lymph nodes (LN) at 0.5 h, but this process was arrested by 3 h after injection. Ricin was successfully targeted to lymphoid tissue as evidenced by a 4-fold increase in ricin-associated radioactivity in LN, a 10-fold increase in the Peyers patches, a doubling in the spleen and a 35% reduction of radioactivity in the liver compared with free ricin. Nevertheless this represented a considerable shortfall in the expected targeting efficiency. The main problem was found to be high in vivo elution of ricin from TDL (70% within 0.5 h of i.v injection). This and other aspects relevant to maximising targeting efficiency are discussed.


Br. J. Cancer (1991), 63, 699–704 © Macmillan Press Ltd., 1991

### Lymphocyte targeted ricin as a potential therapy for lymphoid malignancy. I. Targeting efficiency

C.S. Ramsden, M.T. Drayson & E.B. Bell

*Immunology Group, Department of Cell and Structural Biology, Medical School, University of Manchester, M13 9PT, UK.*

**Summary** Lymphocytes were studied as vehicles to target the plant toxin ricin, to lymphoid tissue in rats. Ricin-loaded thoracic duct lymphocyte (TDL) migrated normally into lymph nodes (LN) at 0.5 h, but this process was arrested by 3 h after injection. Ricin was successfully targeted to lymphoid tissue as evidenced by a 4-fold increase in ricin-associated radioactivity in LN, a 10-fold increase in the Peyers patches, a doubling in the spleen and a 35% reduction of radioactivity in the liver compared with free ricin. Nevertheless this represented a considerable shortfall in the expected targeting efficiency. The main problem was found to be high *in vivo* elution of ricin from TDL (70% within 0.5 h of i.v. injection). This and other aspects relevant to maximising targeting efficiency are discussed.

Ricin, the highly toxic glycoprotein from the castor bean *Ricinus communis*, has been studied extensively at both cellular and molecular levels. Binding of the B-chain glycoprotein to cell surface receptors results in the internalisation and translocation of toxic A chain to its intracellular site of action. Ricin A-chain enzymically inhibits protein synthesis by inactivation of the 60S ribosomal subunit (Sperti *et al.*, 1973). This is achieved by cleavage of an adenine base residue from the 28S ribosomal subunit (Endo *et al.*, 1987).

Ricin has been extensively studied as a potential agent for the treatment of malignancy. When used in laboratory rodents, ricin successfully inhibited growth of Ehrlich ascites tumour cells (Lin *et al.*, 1970; Fodstad *et al.*, 1977). Adjuvant therapy with adriamycin significantly enhanced the lifespan of animals inoculated with leukaemic cells (Fodstad & Pihl, 1980*a*). Ricin treatment suppressed the growth of human tumour cell lines growing as xenografts in athymic mice (Fodstad *et al.*, 1980*b*,*c*). More recently Phase 1 clinical trials have been completed on 54 cancer patients with advanced disease (Fodstad *et al.*, 1984).

In an attempt to improve the efficacy of ricin therapy, various methods have been employed to target ricin to specific sites of action. Immunotoxins have been prepared linking ricin, either as holotoxin or A-chain, to monoclonal antibodies against cell surface antigens. This approach has been utilised *in vitro* to kill contaminating leukaemic cells (Kronke *et al.*, 1985; Press *et al.*, 1986; Katz *et al.*, 1987) from human bone marrow. *Ex vivo* treatment of bone marrow with immunotoxin directed against immunocompetent T lymphocytes has successfully prevented graft-versus-host disease in allogeneic recipients (Vallera *et al.*, 1981; Filipovich *et al.*, 1984).

Another method of targeting is to utilise a migrating population of cells as vehicles to carry material to a particular site in the body. Lymphocytes can be used to carry toxins or radioisotopes to lymphoid tissue (Ford, 1985). Radiolabelled lymphocytes migrate normally for a variable period during which time 80%–90% gain access to lymphoid tissue (Rannie & Donald, 1977; Birch *et al.*, 1986). This method has been successfully employed to deposit radioactive isotopes in spleen and lymph nodes (LN) of normal subjects and patients with lymphoma and leukaemia for diagnostic purposes (Lavender *et al.*, 1977; Wagstaff *et al.*, 1981*a*,*b*). This is currently being developed at the Christie Hospital, Manchester, for therapeutic purposes (Hamilton *et al.*, 1988).

Correspondence: E.B. Bell.
Received 4 July 1990; and in revised form 29 October 1990.

Mammalian cells internalise ricin which is later liberated in a form that is toxic to a second cell population (McIntosh *et al.*, 1984). Sparshott *et al.* (1985) demonstrated that lymphocytes loaded with ricin and trace-labelled with $^{51}Cr$ retained their normal migratory characteristics *in vivo* at early time intervals following injection. These results based on $^{51}Cr$ localisation, suggested that ricin was delivered in high concentrations to lymphoid tissues. The resulting histological changes of lymphoid destruction observed in spleen and LN supported this view. Adoptive transfer of lymphocytes bearing ricin successfully inhibited Moloney-murine sarcoma virus induced tumours *in vivo* (Cerundolo *et al.*, 1987).

In order to assess the efficiency of ricin targeting by lymphocytes the distribution of $^{125}I$-ricin on cells was compared with the distribution of free radioiodinated ricin *in vivo*. The aim of the investigation was to develop means of increasing the ricin concentration in spleen and LN while minimising localisation to the liver and bone marrow.

### Materials and methods

### *Ricin*

The toxin *Ricinus communis* was prepared from the defatted castor bean cake according to the procedures described in detail by Cumber *et al.* (1985). Ricin was kindly supplied by Dr A. Forrester and Professor A.J.S. Davies from the Chester Beatty Cancer Research Laboratory, and at a later stage by Dr P. Thorpe from the Imperial Cancer Research Foundation, London.

### *Radiolabelling ricin holotoxin*

Ricin was radioiodinated by a protocol based on the iodogen method (Johnstone & Thorpe, 1982). Ricin (1.5 mg $ml^{-1}$ in PBS) was added to an iodogen tube containing sufficient powdered lactose, to give a final concentration of 100 mmol. 1 mCi $^{125}I$ (Amersham, Code No IMS 30, specific activity approx 16 mCi $\mu g^{-1}$ of iodine) was added and incubated on ice for 1 h. Free $^{125}I$ and lactose were separated from $^{125}I$-ricin conjugate on a P6 acrylic column equilibrated with 0.6 M sodium acetate buffer. Protein concentration was estimated by spectrometry (LKB Ultrospec 4050) and the specific radioactivity determined.

### *Animals*

Rats of the highly inbred AO or PVG strains ($RT1^u$ and $RT1^c$ respectively) were bred and maintained in the University of Manchester Animal Unit.

*Thoracic duct cannulation*

This was performed by Gowan's method (Ford, 1978). Thoracic duct lymphocytes (TDL) were collected in heparinised Dulbecco's phosphate-buffered saline with added mineral salts either overnight at 0°C or, for not more than 4 h at room temperature.

*$^{51}$Cr-labelled passaged TDL*

TDL collected 'on ice' and subjected to the *in vitro* procedures of radiolabelling, temporarily lose their ability to rapidly migrate into LN (Smith & Ford, 1983). However, these radiolabelled lymphocytes recover *in vivo* following i.v. injection and re-appear in thoracic duct lymph maximally by approximately 18 h later; such cells are referred to as passaged cells and if collected at room temperature and injected within a 4 h period retain their ability to rapidly migrate to LN. Accordingly, an overnight collection of TDL was washed and resuspended in RPMI medium at $10^8$ cells ml$^{-1}$, incubated with $^{51}$Cr (Amersham, Code No CJS 1P, specific activity 250–500 Ci mg$^{-1}$ chromium) at 10 μCi ml$^{-1}$ at 37°C for 1 h. The cells were washed once, resuspended to approximately $5 \times 10^8$ cells ml$^{-1}$ and injected i.v. into thoracic duct-cannulated intermediate rats. $2-3 \times 10^9$ labelled cells (a 20 h collection from four donor rats) were injected into each intermediate animal. Approximately 10% of these cells were recovered in lymph during a 4 h collection the following day.

*Ricin-conjugated TDL*

Since the dual effect of prolonged collection of TDL and low temperature interferes with the recirculation kinetics of TDL (Smith & Ford, 1983), TDL for ricin labelling were collected for a short term (4 h) period at room temperature (RT). TDL was filtered through gauze, washed, counted and resuspended to $10^8$ cells ml$^{-1}$ in RPMI + 10% foetal calf serum (FCS) at 37°C. Ricin was added at 25 μg $10^{-8}$ lymphocytes and incubated for 15 min. Further internalisation of ricin was inhibited by 100 mmol solution of lactose in RPMI + 10% FCS at RT. The cells were washed twice in lactose-RPMI medium and once in RPMI + 10% FCS at RT before final resuspension to $10^8$ cells ml$^{-1}$ for injection.

Cell labelling with radioiodinated ricin enabled quantitation of ricin uptake. The rate of ricin uptake was determined by the regular removal of samples during a 4 h incubation period. An incubation ricin concentration of 25 μg $10^{-8}$ cells was found to be optimal since higher concentrations were known to impair lymphocyte migration to LN, whilst lower concentrations would have necessitated impractically large numbers of TDL to provide a therapeutic dose (Sparshott *et al.*, 1985). Previous studies demonstrated that $^{125}$I-labelled ricin was equal in toxicity to native ricin (Ramsden *et al.*, 1989.

*Release of ricin and chromium-51 from lymphocytes*

$^{125}$I-Ricin-loaded TDL were held *in vitro* at 37°C and ricin release determined. Radioactivity detected in the supernatant was expressed as a percentage of the total radioactivity per sample. In the same way, an estimation of $^{51}$Cr label released from passaged TDL was made. The release of chromium is indicative of cell death and is similar to that of a cytotoxic release assay both *in vitro* and *in vivo* (Rolstad, 1985).

*Tissue distribution of radioactivity*

Rats injected i.v. with free or cell associated $^{125}$I-ricin were anaesthetised, a blood sample obtained by aortic puncture and killed by aortic transection. Individual organs were separated cleanly from adjacent structures, removed, weighed and the amount of radioactivity determined by gamma counting (LKB Ultragamma 1250 dual channel gamma spectrometer). Pulse height analyser facilities for each channel, and automatic comparison for each channel with reference samples allowed discrimination between isotopes ($^{51}$Cr and $^{125}$I) when necessary. Results were expressed as a percentage of the injected dose per gram to reflect the relative concentration in different tissues.

In vitro *studies: inhibition of protein metabolism by ricin*

*Incorporation of $^3$H Leucine (Leu)* 100 μl aliquots of leucine-free culture medium with or without ricin (37.5 μg ml$^{-1}$) were distributed in a 96 well microtitre plate and incubated at 37°C in the presence of 5% $CO_2$. TDL (50 μl of $5 \times 10^6$ ml$^{-1}$ in leucine-free medium) were added to each well. Wells were pulsed with $^3$H Leu (10 μCi ml$^{-1}$, Amersham, Code No TRK 170, Specific activity 70 Ci mmol$^{-1}$ leucine). Cell cultures were harvested at 10 min intervals in the first hour and at 1½, 2, 3, and 5 h thereafter. $^3$H Leu incorporation was measured by scintillation counting a Beckman counter (LS 1801).

### Results

In vitro *uptake of ricin by TDL*

The uptake of ricin by cells was studied by incubating TDL in RPMI + 10% FCS containing $^{125}$I-ricin (25 μg $10^{-8}$ cells) for varying periods. Less than 1% of the ricin was internalised (142 ng to 185 ng $10^{-8}$ cells) by 15 min (Figure 1). Ricin incorporation followed an exponential curve, linear in the first 15 min but levelling off at 30 min; increasing amounts were internalised as late as 4 h.

Since galactose side chains present on serum proteins might compete with cells for ricin binding, TDL were incubated with $^{125}$I-ricin in a medium containing no FCS. Ricin uptake was increased 4–5 fold (800 ng $10^{-8}$ cells, or 3.2% of total). However as indicated later, the absence of protein in the medium had a deleterious effect on lymphocyte migration *in vivo*.

*The distribution of ricin following i.v. injection of ricin-loaded TDL*

The systemic distribution and kinetics of free $^{125}$I-ricin in rats was reported previously (Ramsden *et al.*, 1989). We compared this distribution with that of $^{125}$I-ricin transported *in vivo* by syngeneic TDL. In a number of tissues, notably blood (both cell associated and plasma), heart, muscle and bone marrow, the localisation of ricin, targeted by cells (Table I) or injected free (Ramsden *et al.*, 1989) was similar. Tissues where significant differences in ricin distribution resulted from the transport of ricin on cells are illustrated in Figure 2. At 1 h the localisation of ricin carried to the liver

**Figure 1** Uptake of $^{125}$I-ricin by TDL *in vitro*. The results of two separate experiments (●,*) are shown. Each point is the mean of three samples removed from the incubation mixture.

**Table I** Distribution of $^{125}I$ conjugated ricin loaded lymphocytes 1, 2, 12 and 24 h after injection into recipient animals. Percentage injected dose per gram of tissue (standard deviation in parentheses)

| | *Time (hours)* | | | |
|---|---|---|---|---|
| | *1* | *2* | *12* | *24* |
| Blood cells | 0.57 | 0.57 | 0.44 | 0.16 |
| | (0.11) | (0.09) | (0.18) | (0.10) |
| Blood serum | 1.14 | 0.84 | 0.74 | 0.13 |
| | (0.21) | (0.08) | (0.19) | (0.04) |
| Heart | 0.49 | 0.39 | 0.36 | 0.08 |
| | (0.14) | (0.03) | (0.05) | (0.05) |
| Muscle | 0.22 | 0.13 | 0.15 | 0.04 |
| | (0.15) | (0.02) | (0.01) | (0.01) |
| Small gut | 0.87 | 1.06 | 0.89 | 0.26 |
| | (0.28) | (0.28) | (0.25) | (0.06) |
| Large gut | 0.55 | 0.81 | 0.60 | 0.15 |
| | (0.03) | (0.07) | (0.11) | (0.04) |
| MLN | 5.22 | 6.64 | 4.20 | 0.76 |
| | (0.94) | (0.62) | (0.47) | (0.25) |
| Bone marrow | 5.60 | 5.91 | 2.09 | 0.77 |
| | (1.44) | (1.82) | (0.64) | (0.32) |

**Figure 2** Organ distribution of free ricin (---) or ricin carried by TDL (—) 1, 2, 6 and 12 h following i.v. injection. TDL were labelled with $^{125}I$-ricin (25 μg $10^8$ ml$^{-1}$) and $10^{-8}$ cells injected. Cell-free $^{125}I$-ricin was injected at 1 μg kg$^{-1}$ body weight; data obtained from parallel experiments using the same batch of ricin but published elsewhere (Ramsden *et al.*, 1989). Each point represents the mean ± s.e. of five samples.

by TDL was 30% less than that resulting from i.v.-injection of free ricin. This difference persisted over the next 12 h. There was a doubling of the activity found in the lung at all time intervals, reflecting lymphocyte entrapment within the pulmonary vasculature. Renal localisation following injection of ricin loaded lymphocytes was persistently half that resulting from use of free ricin. In lymphoid tissue we observed a considerable concentration of ricin by targeting with TDL. There was a doubling in the spleen, a 4-fold increase in the cervical LN and 10-fold increase in Peyer's patches.

### *Simultaneous localisation of $^{125}I$-ricin and $^{51}Cr$ by lymphocytes*

In the above experiments the increase in lymphoid localisation of ricin, achieved by lymphocyte targeting, was considerably less than that suggested previously (Sparshott *et al.*, 1985). In these earlier experiments unlabelled ricin was loaded into $^{51}Cr$-labelled TDL and the ultimate distribution of ricin was inferred from the distribution of $^{51}Cr$. In order to clarify the discrepancy a double-labelled experiment was designed to allow for a direct comparison. $^{125}I$-ricin was loaded into passaged $^{51}Cr$-labelled TDL. These double-labelled cells were injected into four groups of five animals each; groups were killed at ½, 2, 12 and 24 h (Figure 3).

$^{51}Cr$ tissue concentrations were used as an indicator of localisation of the TDL. In comparison with published migration kinetics using $^{51}Cr$ TDL (Smith & Ford, 1983), the ricin-loaded TDL localised in spleen and LN almost normally during the first 2 h after injection (Figure 3). The migration pattern differed at subsequent periods in that ricin-loaded TDL failed to be redistributed from spleen to LN, reflecting the toxic effects of ricin on lymphocyte mobility. Figure 3 shows an enormous discrepancy between $^{51}Cr$ and $^{125}I$-ricin distribution. At ½ h spleen and lymph nodes contained high $^{51}Cr$ levels, whereas $^{125}I$-ricin levels were only 50% and 30% of $^{51}Cr$ values, respectively. The level of localisation of $^{51}Cr$ and $^{125}I$ was reversed in the liver (0.9% $^{51}Cr$; 3.7% ricin), bone marrow (4.2% $^{51}Cr$; 8% ricin), blood (0.6% $^{51}Cr$; 1.1% ricin) and kidney (0.3% $^{51}Cr$; 0.7% ricin). Furthermore, while $^{51}Cr$ tended to be retained in lymphoid organs and the liver, $^{125}I$-ricin was rapidly eliminated after 2 h. These results indicate that ricin rapidly dissociated from the lymphocytes after injection, even before they entered lymphoid tissue. Ricin that was transported to lymphoid

**Figure 3** Distribution of radioactivity in AO rats injected with $^{51}Cr$ labelled (---) passaged TDL loaded with $^{125}I$-ricin (—) and killed at ½, 2, 12 and 24 h following i.v. injection. Each point is the mean ± s.e. of five samples.

tissues was retained for up to 2 h but then rapidly released. In contrast $^{51}Cr$ was present for longer periods and released at the time of cell death. It was estimated that about 70% of transported ricin had dissociated from the cells within 0.5 h of injection.

This early release of ricin seriously reduces the efficiency of concentrating ricin in lymphoid tissue. Therefore, we have investigated the early release of ricin and the effect of ricin on TDL *in vitro*.

### In vitro *release of $^{51}Cr$ and $^{125}I$-ricin*

Passaged $^{51}Cr$-labelled TDL, were loaded with $^{125}I$-ricin, washed and incubated in suspension at 37°C. Samples were removed at regular time intervals, centrifuged and both supernatant and cell pellet counted. Release of ricin from TDL in the first 20 min was rapid (Figure 4) and continued but at a slower rate, so that by 120 min 60% had dissociated. The loss of $^{51}Cr$ during this period was minimal; 4% release by 2 h. The *in vivo* release of ricin from TDL by half an hour was 70%, although only 35% release was noted *in vitro*.

### *Effect of ricin on $^{51}Cr$ TDL*

The effect of ricin on $^{51}Cr$-TDL was studied by observing the release of $^{51}Cr$ *in vitro* during 24 h of culture. $^{51}Cr$-labelled TDL were loaded (or not) with ricin, washed, incubated at 37°C and cultures sampled at intervals. The results of two experiments are shown in Figure 5. During the first 2 h less than 5% of $^{51}Cr$ was released from ricin-loaded TDL, but with time the loss of $^{51}Cr$ from these cells increased faster than the release from control cells. By 24 h 85% of $^{51}Cr$ had been released as a result of ricin loading compared with 45–50% from control cells. Maximum $^{51}Cr$ release *in vitro* occurred by 12–24 h correlating closely with the timing of $^{51}Cr$ loss from lymphoid tissues and its appearance in kidney in the *in vivo* situation.

**Figure 4** *In vitro* release of $^{125}I$-ricin and $^{51}Cr$ from $^{51}Cr$-labelled TDL loaded with $^{125}I$-ricin before incubation. Each point is the mean ± s.d. of three samples.

**Figure 5** $^{51}Cr$ release *in vitro* from $^{51}Cr$-labelled TDL loaded with ricin (25 μg $10^{-8}$ cells ml). Different symbols represent two separate experiments. Each point is the mean of three samples.

### *Rapid release of ricin from TDL*

The *in vitro* incubation of TDL loaded with ricin showed two phases of release, an early exponential phase (first 40 min) followed by a slow release phase (Figure 4). We asked whether, by allowing the rapid phase to occur *in vitro* for 40 min before transfer, the selective localisation of ricin to lymphoid tissues by TDL could be improved. Initially we showed that *in vitro* incubated ricin TDL, when washed and incubated *in vitro* for a further 40 min, continued to release ricin at a slow steady rate and did not revert to the rapid release phase. Therefore, $^{125}I$-ricin-loaded TDL were tested *in vivo* after receiving a 40 min incubation step. The $^{125}$-I localisation from standard $^{125}I$-ricin TDL and the 40 min incubated $^{125}I$-ricin TDL was no different (Table II). Allowing an early *in vitro* elution of ricin did not prevent a further elution of ricin *in vivo*, nor improve the targeting of ricin to lymphoid tissue.

### *Effect of ricin on the kinetics of protein synthesis*

In the following experiments the effect of ricin on protein metabolism was examined in an attempt to determine whether there was a correlation with loss of migratory ability, release of ricin, or death of the cell. Protein synthesis was assessed by $^{3}H$-leucine ($^{3}H$-Leu) incorporation. $^{3}H$-Leu was added to cultures of TDL, with or without added ricin (25 μg $ml^{-1}$). Ricin free cultures incorporated $^{3}H$-Leu linearly from 5 min to 5 h. The addition of ricin did not affect $^{3}H$-Leu incorporation during the first 40 min, but by 60 min, $^{3}H$-Leu incorporation had peaked at 70% of the ricin free culture. No further incorporation was detected in the presence of ricin during the subsequent 4 h (Figure 6).

### *Shortening the labelling time and its effect on lymphoid targeting*

In view of the early inhibition of protein synthesis by ricin, the handling time required to load ricin could be critical to the success of ricin targeting *in vivo*. Using a revised protocol in which the handling time was shortened from 60 min to 21 min, we were unable to increase the proportion of $^{125}I$-ricin delivered to LN or spleen in the ½ h period after injection (data not shown).

### Discussion

Lymphocytes provide a powerful tool for targeting materials to lymphoid tissue since, following injection into the bloodstream, they localise in spleen and lymph nodes at a concentration approximately 500 times greater than that averaged over the whole body (Ford, 1985). Ninety percent of this

**Table II** The localisation in AO rats of $^{125}I$-ricin 1 h following i.v. injection of $^{125}I$-ricin loaded TDL prepared by the standard method or incubated *in vitro* for 40 min with removal of 'rapid release' ricin before injection. Values (means ± s.e.) are percentage of injected dose per gram of tissue of three recipients in each group

| | *$^{125}I$-ricin TDL* | |
|---|---|---|
| *Tissue* | *Standard method* | *In vitro incubation* |
| CLN | 5.55 ± (0.35) | 5.95 ± (2.0) |
| Liver | 4.35 ± (0.05) | 3.53 ± (0.4) |
| Spleen | 53.0 ± (5.7) | 50.9 ± (2.5) |

**Figure 6** Cumulative incorporation of $^3$H-Leu by TDL in culture in the absence (control) or the presence of ricin (25 $\mu$g ml$^{-1}$). Each point is the mean ± s.d. of three samples.

targeting is achieved within 1 h of injection, generating lymphoid concentrations 25,000 times that of non-lymphoid tissues such as a muscle. We have clearly failed to achieve this efficiency with ricin targeting by lymphocytes, but have observed some success and identified the problem areas.

The first requisite of lymphocyte targeting is that the cells should be able to take up the material concerned. *In vitro* incubation with ricin results in lymphocyte-uptake of less than 1% of the total ricin. This is not in itself a problem but necessitates that, for clinical use, the ricin be trace-radiolabelled in order to know precisely the dose which will be administered to the recipient.

The second requisite is that ricin should not unduely affect the migration properties of lymphocytes in the first hour or so after injection. The quality of the lymphocytes is important to this area since *in vitro* handling in itself reduces the migratory potential of lymphocytes. Table III illustrates this problem in conventional migration studies using $^{51}$Cr as a trace label. Overnight collection of TDL at 4°C and subsequent *in vitro* manipulations of the trace-labelling process severely inhibit migration to lymph nodes in particular (Smith & Ford, 1983). Where we have had to use $^{51}$Cr labelled cells in our experiments we have first passaged them from blood to lymph of a syngeneic intermediate animal to overcome this problem. When not using $^{51}$Cr labelled cells we have always used freshly collected TDL and avoided cooling below RT or prolonged periods *in vitro* at 37°C which is also detrimental to migratory properties (Smith & Ford, 1983). In clinical studies it is fortunate that lymphocytes freshly-isolated from peripheral blood are equally endowed with good migratory properties (Wagstaff *et al.*, 1981*a*,*b*). It is also clear from the present results, especially Table III, that neither the *in vitro* handling involved in loading lymphocytes with ricin nor the toxicity of ricin itself significantly affect early migration. This is perhaps surprising in the light of the severe and early effects of ricin on protein synthesis by lymphocytes and other cells (Olsnes *et al.*, 1976), but nevertheless indicates that the observed shortfall in targeting efficiency for ricin cannot be accounted for by failure of the lymphocytes themselves to reach lymphoid tissues.

It is the dual labelling experiment with $^{125}$I-ricin $^{51}$Cr-labelled passaged lymphocytes which has elucidated the major problem of early *in vivo* release of ricin from their lymphocytic vehicles before those lymphocytes reach lymphoid tissues (Figure 3). We calculate that this represents a 70% loss of carried ricin into the blood stream in the first half hour after injection and *equally* a 70% reduction in targeting efficiency.

Accordingly we studied elution of ricin from lymphocytes *in vitro* and found the early half hour release of 35% to be only half that observed *in vivo* and to be considerably less thereafter. This higher *in vivo* elution might suggest different mechanisms to those operating *in vitro*. Certainly our attempts to overcome *in vivo* elution by prior washing off of loosely bound ricin with galactose or by permitting a rapidly excreted fraction of ricin to be lost *in vitro* before cell injection were unsuccessful. Cerundolo *et al.* (1987) loaded ricin into T-lymphocytes (either obtained in mass mixed leucocyte-tumour cell cultures or a virus-specific cytotoxic clone) and successfully inhibited the growth of Maloney-murine sarcoma virus induced tumours *in vivo*. Both populations of T-lymphocytes released $^{125}$I-labelled ricin rapidly during the first 30 min *in vitro* and at a constant and slower rate thereafter. The release pattern was quantitatively and qualitatively very similar to that found in the present study for rat TDL. Their ricin loading and subsequent washing procedures were very similar to ours and it is believed that the ricin released represents previously internalised holotoxin. Whether ricin was released faster *in vivo* than *in vitro* in this mouse system was not identified. However the early release of ricin from carrier lymphocytes *in vivo* presents the major obstacle to further reducing systemic toxicity for a given therapeutic effect.

The third requisite of lymphocyte targeting is that following the arrival of lymphocytes in lymphoid tissue they should transfer their contained load of toxin to surrounding cells, preferably with a minimum of spillage into the blood stream. *In vitro* studies have already demonstrated that ricin loaded lymphocytes can transfer a lethal dose of toxin to other cells in coculture (McIntosh *et al.*, 1984). Studies of Sparshott *et al.* (1985) indicate that this also happens with ricin targeting by lymphocytes generating considerable histological damage in lymphoid tissues. Our own histological findings are the subject of a subsequent paper, but it is clear from the current

**Table III** A comparison between the 1 h localisation of TDL collected at 4°C, labelled with $^{51}$Cr and (1) used immediately, (2) used after blood to lymph passage through an intermediate animal to allow full recovery of migration abilities (passaged $^{51}$Cr-TDL), (3) passaged $^{51}$Cr-TDL loaded with $^{125}$I-ricin and used immediately

| | *Overnight TDL** | | *Passaged TDL* | | *Ricin-loaded passaged TDL* | | | |
|---|---|---|---|---|---|---|---|---|
| Localisation | (Cell label) | | (Cell label) | | (Cell label) | | (Ricin label) | |
| Isotope | $^{51}$Cr | | $^{51}$Cr | | $^{51}$Cr | | $^{125}$I | |
| Time after TDL injection (h) | ½ | 2 | ½ | 2 | ½ | 2 | ½ | 2 |
| Liver | 1.2† | 1.1 | 1.0 | 0.6 | 0.9 | 1.0 | 3.7 | 3.0 |
| Spleen | 85 | 105 | 103 | 88 | 110 | 115 | 54 | 39 |
| CLN | 8.2† | 9.4 | 36 | 59 | 23 | 29 | 6.8 | 6.9 |

*Rannie & Donald, (1977). †Values are percent injected dose per gm of tissue.

results that ricin is released from ricin loaded lymphocytes albeit mostly at an early stage and systemically and not in lymphoid tissue. The timing of that release is not particularly related to the cessation of protein synthesis or to the final death of the cells which, by $^{51}$Cr studies both *in vitro* and *in vivo*, probably occurs around 12 h after ricin loading. Ricin which is carried into lymphoid tissues on carrier lymphocytes reaches there within an hour of injection. Subsequent loss of that ricin from lymphoid tissues occurs with identical kinetics to those for free ricin (Figure 2). Whether this represents direct loss from carrier lymphocytes into the blood stream or includes an intermediate passage through surrounding cells is uncertain but the similarity of the kinetics suggests the former alternative to be quantitatively more important.

This work illustrates many of the potential pitfalls in using lymphocytes to target materials in general and ricin in particular. We have identified the main reasons for the shortfall in potential efficiency of lymphocyte targeted ricin, though unfortunately have not been able to overcome the problems involved. Despite these limitations, distribution studies demonstrated successful ricin targeting to lymphoid tissue, with a 4-fold increase in ricin delivered to LN, a 10-fold increase in the Peyers patches, a doubling in the spleen and a 35% reduction of ricin in the liver as compared to free ricin. Ricin is an effective anti-tumour agent in both animal and human tumours and lymphocyte targeting potentially provides a method of treating lymphoid malignancies or micrometastatic lymph node spread from solid tumours.

This work was supported by the Cancer Research Campaign and in part by an MRC Programme Grant No G972/456B. We are grateful for discussion and helpful advice from A.J.S. Davies, J.A. Forrester, D.P. McIntosh and P.E. Thorpe. The ricin was a kind gift from J.A. Forrester and A.J.S. Davies, Chester Beatty Cancer Research Laboratory, London and from P.E. Thorpe, Imperial Cancer Research Foundation, London.

### References

BIRCH, M., SHARMA, H.L., BELL, E.B. & the late FORD, W.L. (1986). The carriage and delivery of substances to lymphatic tissues by recirculating lymphocytes. II: Long term selective irradiation of the spleen and lymph nodes by deposition of Indium 114 m. *Immunology*, **58**, 359.

CERUNDOLO, V., ZANOVELLO, P., MCINTOSH, D., FABBRIS, R., DAVIES, A.J.S. & COLLAVO, D. (1987). Temporary inhibition of Moloney-murine sarcoma virus (M-MSV) induced tumours by adoptive transfer of ricin-treated T lymphocytes. *Br. J. Cancer*, **55**, 413.

CUMBER, A.J., FORRESTER, J.A., FOXWELL, B.M.J., ROSS, W.C.J. & THORPE, P.E. (1985) The preparation of antibody-toxin conjugates. *Methods Enzymol.*, **112**, 207.

ENDO, Y., MITSUI, K., MOTIZUKI, M. & TSURUGI, K. (1987). The mechanism of action of ricin and related lectins on eukaryotic ribosomes. *J. Biol. Chem.*, **26**, 5908.

FILLIPOVICH, A.H., VALLERA, D.A., YOULE, R.J., QUINONES, R.A., NEVILLE, D.M. & KERSEY, J.H. (1984). *Ex-vivo* treatment of bone marrow with anti-T cell immunotoxin for prevention of graft versus host disease. *Lancet*, **i**, 469.

FODSTAD, Ø., OLSNES, S. & PIHL, A. (1977). Inhibitory effect of abrin and ricin on the growth of transplantable murine tumours and of abrin on human cancers in nude mice. *Cancer Res.*, **37**, 4559.

FODSTAD, Ø. & PIHL, A. (1980*a*). Synergistic effect of adriamycin and ricin on L1210 leukaemic cells in mice. *Cancer Res.*, **40**, 3735.

FODSTAD, Ø., AASS, N. & PIHL, A. (1980*b*). Assessment of tumour growth and of response to chemotherapy of human melanomas in athymic, nude mice. *Br. J. Cancer*, **41**, 146.

FODSTAD, Ø., AASS, N. & PIHL, A. (1980*c*). Response to chemotherapy of human, malignant melanoma xenografts in athymic, nude mice. *Br. J. Cancer*, **41**, 453.

FODSTAD, Ø., KVALHEIM, G., GODAL, A. & 4 others (1984). Phase 1 study of the plant protein ricin. *Cancer Res.*, **44**, 862.

FORD, W.L. (1978). The preparation and labelling of lymphocytes. Chapt 23. In *Handbook of Experimental Immunology*. Weir, D.M. (ed.) 3rd edn. Blackwell, Edinburgh.

FORD, W.L. (1985). Exploiting lymphocyte traffic to deliver radioactivity or ricin to lymphatic tissue. *Adv. Exp. Med. Bio.*, **186**, 675.

HAMILTON, D., COWAN, R.A, SHARMA, H. & 5 others (1988). The behaviour of autologous Indium 114 m labelled lymphocytes in patients with lymphoid cell malignancy. *J. Nucl. Med.*, **29**, 485.

JOHNSTONE, A. & THORPE, R. (1982). *Immunochemistry In Practice*, Section 5.2:104–112. Blackwell Scientific Publications.

KATZ, F.E., JANOSSY, G., CUMBER, A. & 4 others (1987). Elimination of T cells from human peripheral blood and bone marrow using a cocktail of three anti-T cell immunotoxins. *Br. J. Haematol.*, **67**, 407.

KRONKE, E.M., DEPPER, J.M., LEONARD, W.J, VITETTA, E.S., WALDMAN, T.A. & GREEN, W.C. (1985). Adult T cell Leukaemia: a potential target for ricin A chain immunotoxin. *Blood*, **65**, 1416.

LIN, J.Y., TSERING, K.Y., CHEN, C.C., LIN, L.T. & TUNG, T.C. (1970). Abrin and ricin: new anti-tumour substances. *Nature*, **227**, 292.

LAVENDER, J.P., GOLDMAN, J.M., ARNOT, R.N. & THAKUR, M.L. (1977). Kinetics of indium-111 labelled lymphocytes in normal subjects and patients with Hodgkin's disease. *Br. Med. J.*, **2**, (6090), 797.

MCINTOSH, D.P., EDWARDS, D.C. & DAVIES, A.J.S. (1984). Transfer of ricin toxicity by spleen cells. *Toxicon.*, **22**, 293.

OLSNES, S., SANDVIG, K., REFSNES, K. & PIHL, A. (1976). Rate of different steps involved in the inhibition of protein synthesis by the toxic lectins abrin and ricin. *J. Biol. Chem.*, **251**, 3985.

PRESS, O.W., VITETTA, E.S., FARR, A.G., HANSEN, J.A. & MARTIN, P.J. (1986). Evaluation of ricin A chain immunotoxins directed against human T cells. *Cell Immunol.*, **102**, 10.

RAMSDEN, C.S., DRAYSON, M.T. & BELL, E.B. (1989). The toxicity, distribution and excretion of ricin holotoxin in rats. *Toxicology*, **55**, 161.

RAMSDEN, C.S., DRAYSON, M.T. & BELL, E.B. (1991). Lymphocyte targeted ricin as a potential therapy for lymphoid malignancy. II. A comparison of the toxicity of free and lymphocyte-targeted ricin. (Submitted).

RANNIE, G.H. & DONALD, K.J. (1977). Estimation of migration of TDL to non-lymphoid tissues. A comparison of the distribution of radioactivity at intervals following IV transfusion of cells labelled with $^{3}$H, $^{14}$C, $^{75}$Se, $^{99m}$Yc, $^{125}$I and $^{51}$Cr in the rat. *Cell Tissue Kinet.*, **10**, 523.

ROLSTAD, B., FOSSUM, S., BAZIN, H. & 4 others (1985). The rapid rejection of allogeneic lymphocytes by a non adaptive cell-mediated mechanism (NK activity). *Immunology*, **54**, 127.

SMITH, M.E. & FORD, W.L. (1983). The recirculating lymphocyte pool of the rat: systematic description of the migratory behaviour of recirculating lymphocytes. *Immunology*, **49**, 83.

SPARSHOTT, S.M., FORRESTER, J.A., MCINTOSH, D.P., WOOD, C., DAVIES, A.J.S. & FORD, W.L. (1985). The carriage and delivery of substances to lymphatic tissue by recirculating lymphocytes. I. The concentratoin of ricin in lymphocyte traffic areas. *Immunology*, **54**, 731.

SPERTI, S., MONTANERO, L., MATTIOLLI, A. & STIRPE, F. (1973). Inhibition by ricin of protein synthesis *in vitro*; 60S ribsomal subunit as the target of the toxin. *Biochem J.*, **136**, 813.

VALLERA, D.A., SODERLING, C., CARLSON, G. & KERSEY, J.H. (1981). Bone marrow transplantation across major histocompatibility barriers in mice. Effect of elimination of T cells from donor grafts by treatment with monoclonal anti-Thy 1.2 plus complement or antibody alone. *Transplantation*, **31**, 218.

WAGSTAFF, J., GIBSON, C., THATCHER, N. & 4 others (1981*a*). A method for following human lymphocyte traffic using indium 111 oxine labelling. *Clin. Expt. Immunol*, **43**, 435.

WAGSTAFF, J., GIBSON, C., THATCHER, N., FORD, W.L., SHARMA, H. & CROWTHER, D. (1981*b*). Human lymphocyte traffic assessed by indium 111 oxine labelling: clinical obervations. *Clin. Exp. Immunol.*, **43**, 443.